# Levels of health literacy among people living with HIV in outpatient care: a cross-sectional study from Denmark

**DOI:** 10.1186/s12981-023-00562-8

**Published:** 2023-08-26

**Authors:** Lotte Ørneborg Rodkjaer, Merete Storgaard, Nanna Toxvig Sørensen, Liv Marit Valen Schougaard

**Affiliations:** 1 Department of Infectious Diseases, Aarhus University Hospital, Aarhus, Denmark; 2 Department of Clinical Medicine, Aarhus University, Aarhus, Denmark; 3 Research Centre for Patient Involvement, Central Denmark Region, Aarhus University, Aarhus, Denmark; 4 Center for Patient-reported Outcomes, AmbuFlex, Gødstrup Hospital, Herning, Denmark

**Keywords:** Human immunodeficiency virus, Health literacy, Outpatient clinics, Patient involvement

## Abstract

**Background:**

Low health literacy (HL) among people living with HIV (PLWHIV) encounter more disease related complications, more difficulty understanding health-related information and low adherence. Considering that, the HL levels among PLWHIV needs to be further investigated. The objective of this study was to investigate the levels of HL and patient involvement among PLWHIV in an outpatient clinic in Denmark. A second objective was to examine differences in HL levels across socio-demographic characteristics.

**Methods:**

In 2019, a population of 682 PLWHIV from a Danish outpatient hospital clinic were enrolled in cross-sectional study. Patients who had a digital postbox received an electronic questionnaire including following domains; health literacy, patient involvement, and socio-demographic status. Health literacy was measured using the Health Literacy Questionnaire (HLQ) through scores on three subscales: social support for health (HLQ4), engaging with healthcare providers (HLQ6), and understanding health information (HLQ9). An unpaired t-test was used to investigate mean differences in the HLQ scores across socio-demographic variables.

**Results:**

A total of 338 (55%) patients responded to the questionnaire. The included participants demonstrated high levels of HLQ4 (mean = 4.2) and HLQ6 (mean = 4.2), but lower for HLQ9 (mean = 2.9). In total 70–80% reported being involved in decisions about their health. We found a positive association between high level of HL (HLQ9) and living with a partner and higher levels of HL (HLQ4, HLQ6, and HLQ9) and employment.

**Conclusion:**

PLWHIV in a Danish out-patient care population were found to have high levels of HL despite differences in demographic characteristics. Further research is needed to examine the levels of HL among non-responders to develop HL approaches and strategies to meet the needs of individuals with different HL skills.

## Background

Due to advances in HIV treatment, individuals infected with the virus now live longer if they are on effective antiretroviral therapy (ART) but require lifelong treatment and continual check-ups to avoid disease progression [1, 2]. The overall management of PLWHIV is multifaceted, with several international guidelines to ensure optimal care is provided [3]. Studies focusing on health literacy (HL) interventions for PLWHIV that focus on adherence, treatment-related skills, and HIV-related knowledge show that HL significantly impacts on health-related outcomes among this population. This is closely associated to socioeconomic status and education, thereby increasing the risks of low HL among those most vulnerable [4].Sufficient HL may be an important precondition for enhancing the health of PLWHIV .and low HL encounter more disease-related complications, more difficulty understanding health-related information and low adherence to HIV treatment [5].

HL is defined as “the combination of personal competencies and situational resources needed for people to access, understand, appraise, and use information and services to make decisions about health. It includes the capacity to communicate, assert and act upon these decisions” [6]. The complexity of the healthcare system increases the demands on patients understanding, accessing, and utilizing health information [7], and aspects of HL represent some of what patients need to navigate the system [8]. Patients’ ability to understand information and navigate the healthcare system are prerequisites for being involved in their own healthcare. Patients are more and more expected to be responsible of their health and be involved in decisions about it, and person-centered care [9] has become an integral part of healthcare policy. Patient involvement is both a legal requirement and a key component of service delivery for healthcare in Denmark [10] and increasing patient involvement through national quality improvement programs is an explicit goal [11]. This is partly because of ethical requirements to properly involve patients in decision-making about their own health, and because of the growing evidence that patient involvement has several benefits [12] such as decrease in hospital admissions, improved effectiveness, efficiency, and quality of health services, and increased quality of life [13]. The need for HL skills increases simultaneously with the massive health information available and societal demands for individuals to be active and involved in maintaining their health to prevent illness [8]. Therefore, it is important to focus on a broad understanding of the complexity of HL, as PLWHIV are on lifelong treatment and need to be checked regularly at the hospital. Comparing HL levels across populations has some challenges due to different definitions, instruments and methodologies being used and increasingly recognized as a multidimensional issue. A population-based study from Denmark [14] showed that 10–20% of the general population found managing key HL tasks difficult. People with long-term conditions reported more difficulties in understanding health information and engaging with health professionals. Perceived HL difficulties were markedly higher in people with lower incomes and educational levels, who live alone, and who are of non-Danish ethnicity. A Danish study from 2020 revealed that despite a relatively highly educated population, inadequate HL was prevalent [15].

To our knowledge, no studies addressing HL have been conducted among PLWHIV in a Danish context, nor on levels of HL among PLWHIV. The primary objective of this study was to investigate levels of HL among Danish PLWHIV in outpatient follow-up in terms of social support for health, engaging with healthcare providers, and understanding health information. The secondary objective was to examine differences in Health Literacy Questionnaire (HLQ) scores across socioeconomic characteristics and relevant dimensions of HL. Our hypothesis was that PLWHIV with low socioeconomic status had a lower level of HL compared to PLWHIV with high socioeconomic status.

## Methods

### Study design and participants

In February 2019, we designed a cross-sectional study involving PLWHIV at the Department of Infectious Diseases at Aarhus University Hospital, Denmark. PLWHIV aged ≥ 18 years with consultations scheduled at the outpatient clinic and access to digital postbox were invited to participate. They received a link to an electronic questionnaire on the AmbuFlex system [16, 17] via a secure digital postbox or “E-box” hosted within Denmark. Patients without access to the digital postbox were unable to respond to the questionnaire. Non-responders received a reminder after two weeks. The questionnaire contained questions regarding socio-demographic factors, HL, and patient involvement.

### Socio-demographic factors

The following variables were recorded: gender, age, national origin, cohabitation, employment, education, year of diagnosis, and current medication adherence. Adherence was assessed by asking about missed doses over the preceding four days: “During the past four days, on how many days have you missed taking all your doses?” with the following response categories: “none”, “one day”, “two days”, “three days”, “four days” [18].

### Health literacy

The HLQ [19] is a 44-item questionnaire with nine subscales that each measure an aspect of the multidimensional construct of HL. All scales have good psychometric properties [19]. The HLQ has been translated and validated in a Danish setting [20]. HL was measured using the HLQ average scores across items from the following subscales: *4: Social support for health* (five items), *6: Ability to actively engage with healthcare providers* (five items), and *9: Understanding health information well enough to know what to do* (five items). Subscale 4 has a four-point ordinal response scale: 1 “strongly disagree”, 2 “disagree”, 3 “agree”, and 4 “strongly agree”. Subscales 6 and 9 have five-point ordinal response scales: 1 “cannot do”, 2 “very difficult”, 3 “quite difficult”, 4 “quite easy”, and 5 “very easy”. The average scores across all items were calculated for each of the subscales. If items were missing, the mean scores of the other items were used to estimate the scale score. The score was not estimated if more than two items were missing. Together, the three scales reflect distinct core competencies patients need to be able to navigate the healthcare system and participate in shared decision-making. No cut points for the levels of HL were defined on the HLQ mean scores as this would have been an arbitrary choice [19]. A higher score indicated a higher level of HL. Low scores on the HLQ4 scale suggested patients who were alone and unsupported in managing their health; low scores on the HLQ6 scale suggested patients who were passive in their approach to healthcare and did not proactively seek information; and low scores on the HLQ9 scale suggested patients who had problems understanding any written health information or instructions about treatments or medications [19].

Use of the HLQ was licensed by Deakin University, Australia prior to the data collection.

### Patient involvement

Five statements about patients’ notions of involvement in healthcare were developed in Denmark by DEFACTUM Social & Health Service and Labour Market [21]: 1 “The healthcare professionals asked questions about my own experiences with my disease”, 2 “I talked to the healthcare professionals about the questions and concerns that I had”, 3 “The healthcare professionals invited me to ask questions and talk about my concerns”, 4 “I was consulted when decisions about my plans were made”, and 5 “I talked adequately to the healthcare professionals about how I manage my condition”. All five items have the following response categories: “Not at all”, “To a lesser extent”, “To some extent”, “To a great extent”, and “To a very high degree”.

### Ethics

The study was approved by the regional committees on health research ethics and the Danish Data Protection Agency (Reg.nr.1-16-02-952-17). Informed consent was obtained via the link they received filling out the questionnaire, and data were handled anonymously and confidentially at all stages of the research in accordance with the Helsinki Declaration [22].

### Statistical analysis

Analyses were performed using STATA statistical software version 15.1. The mean differences of the HLQ scores and 95% confidence intervals (CIs) were calculated using an unpaired t-test between socio-demographic groups (gender, age, national origin, cohabitation, employment, education, year of diagnosis, and medication adherence). Normality was not assumed; thus, CIs were estimated using the bootstrap method with 1000 replications [23].

Statistical significance was defined as *p* < 0.05. Descriptive analyses including numbers, percentages, means, and standard deviations (SDs) were used to illustrate the socio-demographic variables and levels of HL and patient involvement. If data were not normally distributed, medians and interquartile ranges (IQRs) were also reported.

## Results

### Patient characteristics

**Fig. 1:**
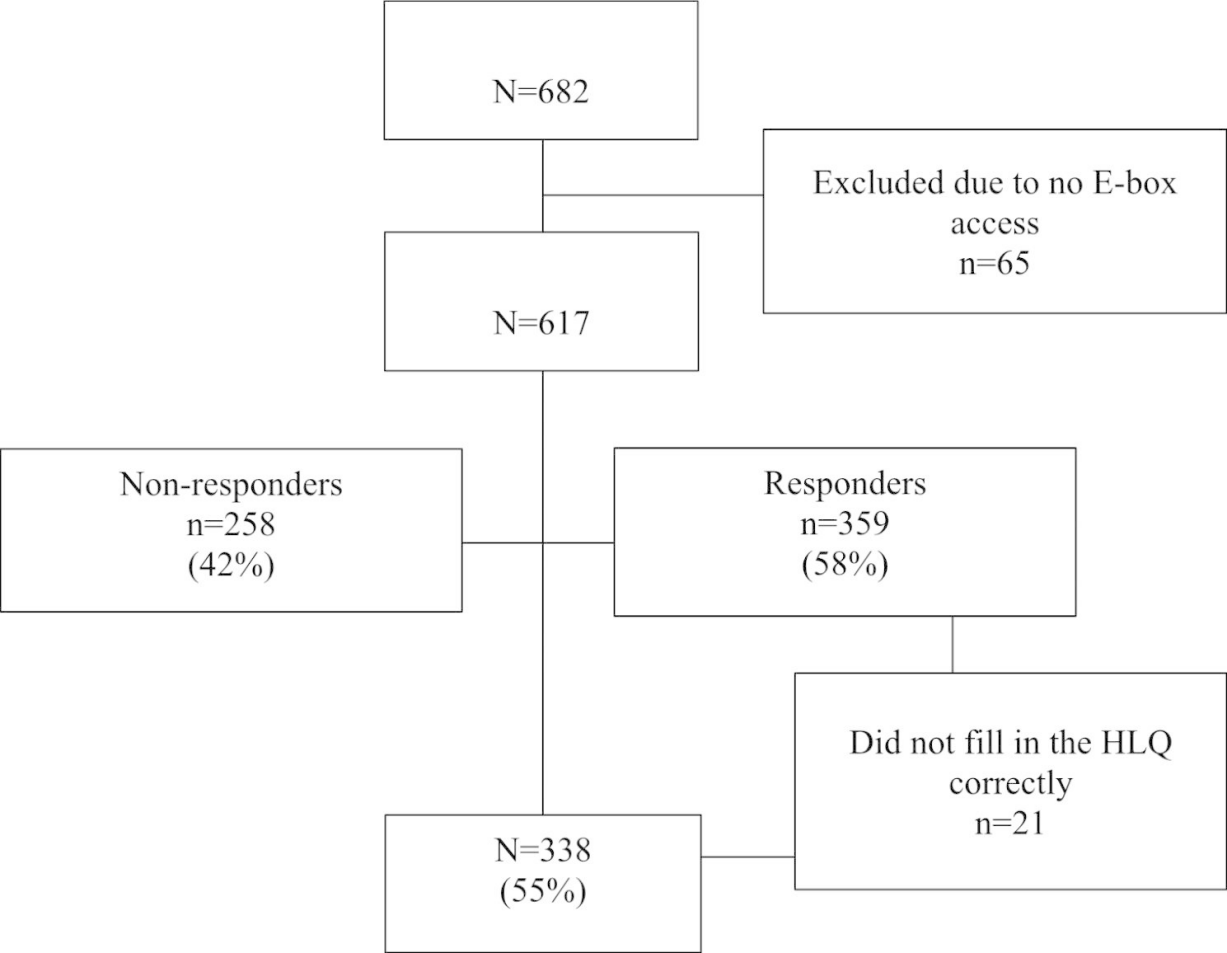
Flowchart of respondents and non-respondents among 682 PLWHIV

Of the overall population of 682 eligible patients, 65 (10%) were excluded due to inability to access the E-box, and 258 (42%) did not respond to the invitation to participate. Of the remaining 359 (58%) individuals, 21 were excluded because they did not complete the HL questionnaire correctly. Consequently, a total of 338 PLWHIV were included in the study. Recruitment of study participants is shown in Fig. 1.

Respondent characteristics are listed in Table 1. A total of 70% of the participants were male, 75% were under 60 years of age, and 72% were Danish. More than half were married or living with a partner (55%), 49% were in full-time employment, 69% had more than 10 years of education, and only 15% had no further education at all. Moreover, 76% had received their HIV diagnosis after 1996, and 96% had not missed any doses of ART.

**Table 1:** Demographic characteristics of 338 PLWHIV

	N = 338	%
*Gender*		
Male	238	70
Female	100	30
*Age*		
18–39	53	16
40–49	96	28
50–59	104	31
60–69	56	17
70+	29	8
*National origin*		
Danish	244	72
Other	94	28
*Cohabitation*		
Married/living with a partner	187	55
Living alone	151	45
*Employment*		
Full-time	167	49
Part-time	46	14
Unemployment/sickness/disability support benefits	40	12
Retired	85	25
*Education*		
Primary or elementary school < 7 years	40	12
Primary or elementary school 8–9 years	52	15
Primary or elementary school 10 years	83	25
High school graduation	123	36
*Further education*		
No further education	51	15
A few years of higher education (1/2 year – 3 years)	115	34
More years of higher education (3–4 years)	77	23
Completed higher education (more than 4 years)	52	15
Other	43	13
*Year of HIV diagnosis*		
Before 1996	82	24
After 1996	256	76
*During the past four days, on how many days have you missed taking all your doses?*		
None	317	94
1–4 days	21	6

### HL scores

Table 2 shows the mean and median self-reported HLQ scores for the 338 participants. These were high for HLQ4 (mean = 4.2) and HLQ6 (mean = 4.2), but lower for HLQ9 (mean = 2.9).

**Table 2:** Self-reported HLQ scores for 338 participants

	Total (%)
	N = 338
Social support for health (HLQ4)	
Mean (SD)	4.2 (0.77)
Median (IQR)	4.4 (4–5)
Ability to actively engage with healthcare providers (HLQ6)	
Mean (SD)	4.2 (0.68)
Median (IQR)	4.3 (3.8 − 4.8)
Understanding health information well enough to know what to do (HLQ9)	
Mean (SD)	2.9 (0.72)
Median (IQR)	3 (2.6–3.4)

Abbreviations – SD: standard deviation, IQR: interquartile range, HLQ: Health Literacy Questionnaire

### HL levels across socio-demographic factors

Table 3 presents the mean scores for the HLQ scales stratified by different socio-demographic variables. Participants living alone had statistically significant lower HLQ9 scores compared to participants living with a partner, with a mean difference of 0.27 (95% CI 0.12–0.42), *p* = 0.021. Participants in employment also had statistically significant higher HLQ scores compared to those not in employment, with an HLQ4 mean difference of 0.20 (95% CI 0.03–0.38), *p* = 0.012, an HLQ6 mean difference of 0.19 (95% CI 0.04–0.33), *p* < = 0.001, and an HLQ9 mean difference of 0.25 (95% CI 0.09 to 0.40), *p* = 0.002.

**Table 3:** Associations between HLQ scores and patient characteristics (N = 338)

		HLQ4	HLQ6	HLQ9
**Gender**, **mean (SD)**				
Female	(n = 100)	4.2 (0.77)	4.1 (0.75)	3.0 (0.61)
Male	(n = 238)	4.3 (0.76)	4.2 (0.66)	2.9 (0.75)
Mean difference (95% CI)		0.13 (-0.04–0.731)	0.05 (-0.11–0.23)	− 0.06 (-0.22–0.09)
**Age, mean (SD)**				
≤ 50	(n = 149)	4.2 (0.77)	4.1 (0.71)	2.9 (0.70)
≥ 51	(n = 189)	4.3 (0.76)	4.2 (0.66)	2.9 (0.73)
Mean difference (95% CI)		0.08 (-0.07–0.24)	0.12 (0.02–0.26)	0.01 (-0.13–0.16)
**National origin, mean (SD)**				
Danish	(n = 244)	4.3 (0.73)	4.1 (0.77)	3.0 (0.73)
Other	(n = 94)	4.16 (0.84)	4.2 (0.64)	2.8 (0.68)
Mean difference (95% CI)		0.13 (-0.05–0.3)	0.16 (-0.16–0.33)	0.14 (-0.03–0.3)
**Cohabitation, mean (SD)**				
Married/living with a partner	(n = 187)	4.2 (0.76)	4.2 (0.71)	3.0 (0.62)
Living alone	(n = 151)	4.2 (0.78)	4.2 (0.64)	2.7 (0.78)
Mean difference (95% CI)		0.04 (0.13–0.20)	0.02 (0.12–0.16)	0.27 (0.12–-0.42) *
**Education, mean (SD)**				
≤ 10 years	(n = 92)	4.2 (0.81)	4.1 (0.76)	2.8 (0.70)
≥ 11 years	(n = 246)	4.2 (0.75)	4.2 (0.65)	2.9 (0.72)
Mean difference (95% CI)		− 0.06 (-0.25–0.12)	0.10 (-0.06–0.28)	0.04 (-0.13–0.20)
**In employment, mean (SD)**				
Yes	(n = 213)	4.3 (0.73)	4.3 (0.76)	3.0 (0.68)
No	(n = 125)	4.1 (0.81)	4.0 (0.72)	2.7 (0.75)
Mean difference (95% CI)		0.20 (0.03–0.38) *	0.19 (0.04–0.33) *	0.25 (0.09–0.40) *
**Year of HIV diagnosis, mean (SD)**				
≤ 1996	(n = 82)	4.2 (0.79)	4.3 (0.64)	3.0 (0.79)
≥ 1997	(n = 256)	4.2 (0.76)	4.2 (0.70)	2.9 (0.70)
Mean difference (95% CI)		0.06 (-0.19–0.20)	− 0.14 (-0.30–0.01)	− 0.04 (-0.23–0.14)
**Doses missed during the last four days, mean (SD)**				
None	(n = 317)	4.3 (0.77)	4.2 (0.69)	2.9 (0.72)
1–4	(n = 21)	4.2 (0.75)	4.2 (0.66)	2.9 (0.65)
Mean difference (95% CI)		0.06 (-0.26–0.38)	0.01 (-0.27–0.29)	− 0.06 (-0.34–0.22)

*p < 0.05

HLQ4 = Social support for health

HLQ6 = Ability to actively engage with healthcare providers

HLQ9 = Understanding health information

### Level of patient involvement

Table 4 presents the levels of patient involvement. The results show that more than half of the patients (76%) replied that they had talked to the healthcare professionals about their issues and concerns to a very high or high degree. More than half of the patients felt the healthcare professionals asked questions about their experiences with their disease (62%) and invited them to ask questions and talk about their concerns (57%). Furthermore, 74% reported that they had talked to the healthcare professionals about the best way to manage their condition to a very high or high degree, and 71% reported that they were consulted when making decisions about what was going to happen to a very high or high degree.

**Table 4:** Patient involvement among 338 PLWHIV

	N = 338	%
*The health professionals asked for my own experience with my illness/condition*		
Very much	77	23
A lot	132	39
Somewhat	88	26
A little	25	7
Not at all	16	5
*I talked to the healthcare professionals about the issues or concerns I had*		
Very much	107	32
A lot	148	44
Somewhat	61	18
A little	15	4
Not at all	7	2
*The healthcare professionals urged me to ask questions or talk about my concerns*		
Very much	87	26
A lot	106	31
Somewhat	101	29
A little	22	7
Not at all	22	7
*I was consulted when making decisions about what was going to happen*		
Very much	115	34
A lot	125	37
Somewhat	71	21
A little	19	6
Not at all	8	2
*I have had adequate conversations with the healthcare professionals*		
*about how best to manage my illness/condition*		
Very much	103	30
A lot	148	44
Somewhat	67	20
A little	15	4
Not at all	5	2

## Discussion

This study investigated levels of HL in terms of social support for health, engaging with healthcare providers, and understanding health information among PLWHIV in Denmark. Overall, the study found that PLWHIV had high levels of HL despite differences in their demographic characteristics. However, we found that patients living alone or who were not in employment reported lower levels of HL compared to those living with a partner or who were in employment. Patients also reported a high degree of involvement in interactions with the healthcare professionals at the outpatient clinic.

Studies show that poor HL is a social barrier to accessing healthcare services and obtaining suitable medical treatment among patients living with HIV and is influenced by low socioeconomic status [24, 25]. The review by Palumbo [25] found that levels of HL among PLWHIV influence their life situation and health status. Especially persons from inadequately served ethnic minority groups, people with refugee backgrounds, substance abuse, mental illness or homelessness. In this study, we found that PLWHIV from other countries reported lower scores on HLQ4 and HLQ9 compared to their counterparts with a Danish background; however, the differences were small and not statistically significant. In our study, participants living with a partner had higher HLQ9 scores (understanding written information) compared to participants living alone. Associations between living alone and low HL have been identified in a Danish study population living with cancer [26]. This raises an issue, which has been discussed in the literature, acknowledging that the links between HL and health outcomes are not direct or linear. Rather, they are “mediated” by factors that are themselves indivisible from each individual’s social environment. Studies shows that the HL abilities, skills, and practices of other people in the same social environment influence an individual’s HL. This means that HL is a “distributed resource” within the individual’s social network, which is the situation for people living with a chronic disease such as HIV. Edwards et al. [27] argue that while individuals’ HL may vary within a group, they can overcome personal insufficiencies in their HL skills by merging their efforts. In this way, distributed HL becomes a resource that can mediate the negative consequences of low HL. Our study also showed a statistically significant difference in scores for HLQ4, HLQ6, and HLQ9 between those who were in employment and those who were not. This finding is supported by a Danish study that revealed those who were not working and who were receiving economic public support were more likely to demonstrate inadequate HL competencies compared to those who were active in the labor market, when age and socioeconomic factors were taken into account [28]. Another Danish study, involving patients with liver cirrhosis, also found low levels of HL among patients with low levels of education and those living alone [29]. Based on the literature, factors that influence an individual’s level of HL may include: (1) demographic, social, and cultural circumstances (e.g., socioeconomic status, employment, income, social support, culture, and language), and (2) individual characteristics (e.g., age, gender, cultural background, physical abilities, social skills, cognitive skills, earlier experience with illness, and experience of the healthcare system) [7, 30]. One study investigated the impact of HL levels on healthcare utilization in a Danish context in relation to various chronic diseases. It revealed that patients with cardiovascular disease, chronic obstructive pulmonary disease, diabetes, and mental disorders reported lower levels of HL in terms of having more difficulties than the general population in understanding health information and engaging with healthcare professionals [26]. For PLWHIV with access to ART, HIV has become a chronic condition requiring a lifetime dedication to cope with their disease. As such, patients living with HIV have ongoing interactions with the healthcare system and professionals throughout the course of their life. Longer experience of navigating the healthcare system may result in the development of better HL skills. This is a potential reason why our findings indicate that a high proportion of PLWHIV do not find navigating the healthcare system particularly complex, whatever their gender, age, and level of education. This is supported by Dawson-Rose et al. [31], who found that HL is strongly influenced by the relationships PLWHIV develop with their healthcare providers. HL is a complex phenomenon involving access to and use of health information to inform and empower health decisions and behaviors. HL consists of several competencies which are not merely functional competencies and include interactive and critical skills. HL is dynamic, responding to various contextual, individual, and situational factors [7]. The view of HL as a process that is actively influenced by the relationships individuals develop with their healthcare professionals, and by changes during their lives, is supported by Brinkley-Rubenstein et al. [32]. They maintain that HL should be conceptualized and operationalized as a process rather than a static situation where knowledge is given from healthcare professional to the patient. They argue that important relational factors necessary for the development of HL include trust, acknowledging the patient’s multiple social identities, and understanding the range of needs that the patient might have. Individuals’ requirements for health competencies depend on the complexity of their condition and the healthcare they are offered. Their individual and social resources may change during the course of their life. Therefore, participants’ HLQ responses may have been influenced by the complexity of their health needs and the contextual challenges of their disease. This highlights the need for ongoing research, including the development of modified tools to measure HL that are relevant to cope with HIV-related conditions. To improve the quality of future studies, Fernandez-Guiterrez et al. [33] suggest using an HL framework that includes both the functional, interactive, and critical dimensions. Studies have reported that levels of HL influence on knowledge of HIV [34, 35] and are determinants of adherence to HIV treatment [36, 37], and that low levels of HL are associated negatively on patients’ understanding of health information [35]. Our study found high levels of HL and adherence to ART. Other studies report inconsistent findings on the association between HL and medication adherence among people living with HIV [5, 35, 36, 38], which might be due to the many definitions of HL and therefore the diverse scales used to measure it. Based on an understanding of HL as a complex phenomenon which goes beyond the functional [36] and a meta-analysis of the literature, D’Eath et al. [39] observed that the HL intervention literature has remained focused on functional literacy. Due to a lack of agreement on both the definition and measurement of HL, it has contributed to a lack of consensus on the HL, which leads to difficulties in comparing results across studies. In this study, we measured average HLQ scores for the subscales HLQ4, HLQ6, and HLQ9. Overall scores were high for all participants. However, HLQ 9 (understanding written information) scores were lower than those of the Danish background population [20]. Among the 481 participants in the study by Maindal et al. [20], the mean value for HLQ9 was 3.99 compared to 2.9 in our study. This might be explained by the differences in the two cohorts’ national origins: in the 2016 study, 92.9% were Danish; in our study, 72% were Danish and 28% from other countries. In our study, the participants experienced high levels of involvement in their consultation, e.g., they had talked about their issues and concerns, about their experiences with their disease, and how best to manage their condition. Having an ongoing and trusting patient-provider relationship is one of the most important contributors to the health of PLWHIV [31]. Building the necessary trust is an iterative and mutual process that occurs over time [31]. Further involvement in decision-making might also positively affect medication adherence behaviors and survival [40].

### Limitations and strength

Our study had some limitations. Firstly, it was a cross-sectional study and could not preclude any causal conclusion. Secondly, the survey had a modest response rate of 55%, and the absence of information about non-responders affects our knowledge about potential challenges for this population, e.g., patients’ low literacy or issues around engaging with care might lead to selection bias. We know from findings in other Danish studies in patients with epilepsy and rheumatoid arthritis that non-responders and patients without access to a digital postbox had a lower sociodemographic status [41, 42]. Considering that, non-responders in our study might have lower HL, so our results potentially represent an overestimate of HL levels. The ability and motivation to complete a health survey represent an HL competency in themselves; thus, the most vulnerable groups may not have participated in the study. Thirdly, the questionnaire was only available in Danish, limiting participation in the study to patients literate in the language. An equitable and inclusive approach are of major importance in health survey research [43]. To increase an inclusive approach in our study, we should have considered different modes of questionnaire administration and language versions Finally, the study was only conducted in one hospital department. However, to the best of our knowledge, this is the first study addressing HL levels among PLWHIV in a Danish context. A strength of the study is that it was based on three of the nine dimensions of the HLQ and used a validated and culturally adapted questionnaire. The three subscales were selected because they cover two distinct and central dimensions of HL, providing valuable insights into the HL challenges facing individuals with chronic disease. However, the selection of only three scales might result in underrepresentation of the complexities involved in HL.

## Conclusions

PLWHIV in Denmark were found to have generally high levels of HL despite differences in demographic characteristics. Further research is needed to examine the levels of HL amongPLWHIV who did not respond to the survey in order to develop HL approaches and strategies to meet the needs of individuals with a range of HL skills.

## Data Availability

Not applicable.

## Abbreviations

HL: Health literacy
ART: antiretroviral therapy
Cis: confidence intervals
SDs: standard deviations
IQRs: interquartile ranges
